# Chimeric Capsid Proteins Impact Transduction Efficiency of Haploid Adeno-Associated Virus Vectors

**DOI:** 10.3390/v11121138

**Published:** 2019-12-09

**Authors:** Zheng Chai, Xintao Zhang, Amanda Lee Dobbins, Ellie Azure Frost, R. Jude Samulski, Chengwen Li

**Affiliations:** 1 Gene Therapy Center, Department of Pediatrics, University of North Carolina at Chapel Hill, Chapel Hill, NC 27599, USA; zchai@email.unc.edu (Z.C.); xintao1@email.unc.edu (X.Z.); amanda_dobbins@med.unc.edu (A.L.D.); azure@live.unc.edu (E.A.F.); rjs@med.unc.edu (R.J.S.); 2 Department of Pharmacology, University of North Carolina at Chapel Hill, Chapel Hill, NC 27599, USA; 3 Department of Pediatrics, University of North Carolina at Chapel Hill, Chapel Hill, NC 27599, USA; 4 Carolina Institute for Developmental Disabilities, University of North Carolina at Chapel Hill, Chapel Hill, NC 27510, USA

**Keywords:** AAV, gene therapy, enhanced transduction, haploid vector

## Abstract

Our previous studies have demonstrated that haploid AAV vectors made from capsids of two different serotypes induced high transduction and prevented serotype-specific antibody binding. In this study, we explored the transduction efficiency of several haploid viruses, which were made from the VP1/VP2 of one serotype and VP3 of another compatible serotype. After systemic injection of 2 × 10^10^ vg of AAV vectors into mice, the haploid AAV vectors, composed of VP1/VP2 from serotypes 8 or 9, and VP3 from AAV2, displayed a two to seven-fold increase in liver transduction compared with those of parental AAV2 vectors. Furthermore, a chimeric AAV2/8 VP1/VP2 with N-terminus of VP1/VP2 from AAV2 and C-terminus (VP3 domain) from AAV8 was constructed, and produced the haploid vector 28m-2VP3 with AAV2 VP3. The haploid 28m-2VP3 vector showed a five-fold higher transduction than that of the vectors composed solely of AAV2 VPs. Remarkably, the 28m-2VP3 vectors also induced a significant increase in transgene expression compared to the vectors composed of AAV8 VP1/VP2 with AAV2 VP3. The results suggest that the difference in the VP1/VP2 N-terminal region between AAV2 and AAV8 may allow better “communication” between the VP1/VP2 N-terminus of AAV2 with its cognate VP3. Similarly, the haploid vectors, VP1/VP2 from serotypes 8 or 9 and VP3 from AAV3, achieved higher transductions in multiple tissue types beyond typical tropism compared with those of AAV3 vectors. Consistently, higher vector genome copy numbers were detected in these tissues, indicating that an incorporation of non-cognate VP1/VP2 might influence the cellular tropism of the haploid vectors. However, there was no significant difference or even decreased transductions when compared with those of parental AAV8 or AAV9 vectors. In summary, these studies provide insight into current development strategies of AAV vectors that can increase AAV transduction across multiple tissues.

## 1. Introduction

Adeno-associated virus (AAV) belongs to the family of *Parvoviridae*. It is a small, non-enveloped virus with a single-stranded DNA genome. The AAV genome contains two genes, *rep* and *cap*, which encode the nonstructural *rep* proteins and the structural *cap* proteins, respectively [1]. The AAV virion is an icosahedral capsid that is formed from the assembly of three viral proteins (VP), VP1, VP2, and VP3 in an approximate ratio of 1:1:10. These three viral proteins share overlapping open reading frames and all have the sequence of VP3 protein in common. VP1 and VP2 have N-terminal extension of 202 and 65 additional amino acids of VP3, respectively.

Non-pathogenic AAV, a replication-defective virus, requires a helper virus for efficient replication. As a viral vector, AAV has been used for gene therapy because it is safe and simple. To date, 13 AAV serotypes and more than 100 variants have been isolated and characterized. So far, among 13 serotypes isolated, several serotypes and variants have been applied for clinical trials [2]. As the first characterized capsid, AAV2 has been widely used in gene delivery such as the *Factor IX* gene for hemophilia B and the *RPE 65* gene for Leber congenital amaurosis [3,4,5]. In addition, AAV8 has also been recognized for effective transgene delivery in the liver, and is a lead candidate in clinical trials of hemophilia [6,7,8]. In many pre-clinical studies, AAV9 has demonstrated its ability to cross the blood-brain barrier and achieve widespread central nervous system gene expression [9]. Notably, the US Food and Drug Administration has approved two AAV gene therapy reagents using AAV2 delivering RPE65 for inherited vision loss and AAV9 delivering the *SMN* gene for spinal muscular atrophy [10]. The safety and therapeutic efficacy of AAV vectors has been proven in clinical trials; however, one of the most challenging aspects of AAV vector is its low infectivity, requiring administration of relatively huge numbers of virus genomes. High doses of vectors could induce high levels of immune response against AAV vectors and potential toxicities in patients. Thus, the collective data from clinical trials call attention to exploring effective approaches for enhancing AAV transduction.

In our previous studies, we have demonstrated that enhanced AAV transduction has been achieved using polyploid vectors [11]. Polyploid AAV vector is defined as a vector, which is produced from the co-transfection of capsids from different serotypes parents or mutant serotype parents that results in a wt AAV virion assembled from 60 intact capsomere subunits. For example, haploid AAV vectors were produced by transfection of two AAV helper plasmids (AAV2 and 8) or triploid AAV vectors from three helper plasmids (AAV2, 8, and 9) [11]. These individual polyploid vector virions may be composed of different capsid subunits from different serotypes. For example, haploid AAV2/8, which is generated by co-transfection of AAV2 helper and AAV8 helper plasmids, may produce capsid subunits with a variety of combinations including VP1, VP2, and VP3 derived from AAV2 and AAV8 in one virion for effective transduction. It is impossible to elucidate the mechanism for why the combination of capsid subunits from different AAV serotypes contributes to the enhancement of transduction efficacy. To further understand the enhancement of transduction efficacy observed with these polyploid vectors, chimeric capsid proteins of AAV have been generated in this study.

In this study, we have made a series of constructs for AAV helper plasmids with mutations in the start codons of capsid ORFs, in which only one or two VP were expressed. Additionally, we made chimeric AAV helper constructs in which VP1/2 were derived from two different serotypes (AAV2 and AAV8). We used these constructs to produce a series of haploid AAV vectors and evaluate their transduction efficacy in mice. We found that enhanced transduction was achieved from haploid vectors with VP1/VP2 from serotypes 8 and 9, and VP3 from AAV2, AAV3, or mutant AAV2G9 when compared with that of parental AAV2, AAV3, or AAV2G9. Further, our study showed that haploid AAV vectors made from the chimeric VP1/VP2 capsid with N-terminus from AAV2 and C-terminus from AAV8 and VP3 from AAV2 induced much higher transduction. Our results provide mechanistic insights for designing novel AAV vectors for transduction enhancement in future clinical applications.

## 2. Materials and Methods

### 2.1. Plasmids and Site-Directed Mutagenesis

All of the plasmids that were used to express VP1/2 and VP3 were made by site-directed mutagenesis. Mutagenesis was performed by QuikChange II XL Site-Directed mutagenesis Kit (Agilent, Santa Clara, CA, USA) according to the manual provided in the kit. The chimeric *cap* ORF that contains the N-terminus (1–201 aa) of the AAV2 capsid and C-terminus of the AAV8 capsid was generated by overlapping PCR assay. Then, the fragment was cloned into the *Swa I* and *Not I* sites of pXR. All of the primers in this study are shown in Supplementary Table S1. All of the mutations and constructs were verified by DNA sequencing.

### 2.2. Virus Production

AAV vector was produced by three plasmids co-transfection, as previously described [11]. Briefly, three plasmids (9 μg of AAV transgene plasmid pTR/CBA-Luc, 12 μg of AAV helper plasmid, and 15 μg of Ad helper plasmid pXX6-80) were co-transfected into a 15-cm dish of HEK293 cells. After 60 h, HEK293 cells were collected, lysed, and ultracentrifuged in CsCl gradient. Virus was titrated by quantitative PCR.

### 2.3. In Vitro Transduction Assay

Huh7 and C2C12 cells were infected by AAV vectors with 1 × 10^4^ vg/cell in a 24-well plate. Forty-eight hours post-infection, cells were lysed and measured by a luciferase assay system (Promega, Madison, WI, USA).

### 2.4. Animal Study

Six-week-old female C57BL/6 mice (Jackson Laboratory, Bar Harbor, ME) were used in this study [12]. In accordance with NIH guidelines, the mice were maintained in the UNC Institutional Animal Care and Use Committee (IACUC, IACUC ID: 15-294.0, Approval date: Oct 8th, 2015). The mice were administered 2 × 10^10^ vg of AAV vectors via retro-orbital injection. Luciferase expression was captured using Xenogen IVIS Lumina (Caliper Lifesciences, Waltham, MA, USA) at 1-week post-injection. Bioluminescent pictures were analyzed using Living Image (PerkinElmer, Waltham, MA, USA).

### 2.5. Quantitation of Luciferase Expression in Mouse Tissue

The mice were sacrificed at 4-week post AAV injection. The livers were collected, minced, and homogenized in passive lysis buffer (Promega, Madison, WI, USA). The liver lysates were centrifuged and the supernatant was used to test the luciferase activity. Total protein concentration in the supernatant was measured by the Bradford assay (BioRad, Hercules, CA, USA).

### 2.6. Detection of AAV Genome Copy Number in Mouse Tissue

Total genomic DNA from the mouse tissues, including hearts, livers, lungs, kidneys, muscle, and brains, was isolated by the DNeasy Blood and Tissue Kit (Qiagen, Hilden, Germany. Absolute qPCR assay was performed to measure the luciferase gene and mouse *GAPDH* gene.

### 2.7. Immune-Blot of Heating or pH-Treated AAV Particles

The same amounts of AAV2, H-AAV8-2, AAV82, and 28m-2vp3 viral particles (5 × 10^9^ vg) were treated either at 60 °C, 63 °C, 65 °C, 67 °C, and 70 °C for 10 min or at pH 4, 5, 6, or 7.5 for 10 min. The immune-blot assay was processed as described before [11]. Briefly, the samples were loaded on an NC membrane using a vacuum dot-blotter. The membranes were blocked for 1 hour in 10% milk PBS and then incubated with monoclonal antibody A20, B1, or A1. The membranes were incubated with a peroxidase-coupled goat anti-mouse antibody for 1 h. The signals were captured using Amersham Imager 600 (GE Healthcare Biosciences, Pittsburg, PA, USA).

### 2.8. Statistical Analysis

The data were presented as mean ± SD. Two-group comparisons were applied by the Student *t* test, while three or more group comparisons were tested by *Tukey’s* multiple comparison. *P* values of <0.05 were considered as statistically significant.

## 3. Results

### 3.1. Haploid Vectors with VP3 of AAV2 and VP1/VP2 of Other Serotypes Enhanced the Liver Transduction of AAV2

Our recent study showed that the haploid AAV2/8 viruses made from co-transfection of AAV2 and AAV8 helper plasmids could increase liver transduction efficacy with the appropriate ratio of capsid proteins [11]. However, the capsids of the haploid virus might have many possible combinations of capsid proteins from AAV2 or AAV8 such as AAV2VP1/VP2 + AAV8 VP3, AAV2 VP1 + AAV8 VP2/VP3, AAV2 VP2 + AAV8 VP1/VP3, AAV2 VP3 + AAV8 VP1/VP2. To further explore the potential role of VP1/VP2 in high transduction of haploid virus, we made different helper plasmids that expressed VP1/VP2 of one serotype and VP3 of another serotype by genetic missense mutations of the AAV cap ORF translational start codons. With pXR2 as a template, we mutated the VP1 and VP2 start codons to generate the construct AAV2 VP3 using site-directed mutagenesis with different primers (Supplementary Table S1). Similarly, the start codons, M203L and M211L, were mutated to make the construct AAV8 VP1/VP2 (Supplementary Table S1).

**Figure 1:**
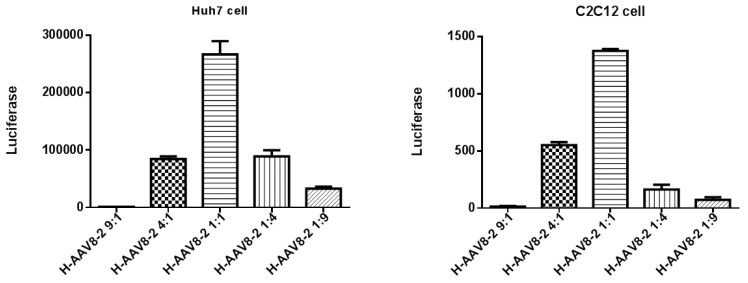
In vitro transduction of the haploid viruses. Huh7 or C2C12 cells were infected with 10^4^ vg/cell of haploid virus. Luciferase assay was performed at 48 h post-transduction. The data represent an average of three separate infections.

To determine the appropriate ratio of capsid proteins for high transduction of haploid virus, we first produced the haploid viruses by co-transfecting two helper plasmids expressing AAV8 VP1/VP2 and AAV2 VP3 at ratios of 9:1, 4:1, 1:1, 1:4, and 1:9. We found that the virus yield was decreased if the amount of one serotype capsid was much more than that of another one for transfection, such as at the ratios 9:1 and 1:9 of AAV8 VP1/VP2 to AAV2 VP3, but there was no significant difference in transduction. When the same amount of haploid viruses (9:1, 4:1, 1:1, 1:4, and 1:9) were used to transduce into either Huh7 or C2C12 cells, the haploid virus designated as H-AAV82 at a ratio of 1:1 achieved the highest transduction efficiency among these haploid viruses (Figure 1). Based on the results, we made all of the haploid viruses with the ratio of 1:1 for following in vitro and in vivo experiments.

**Figure 2:**
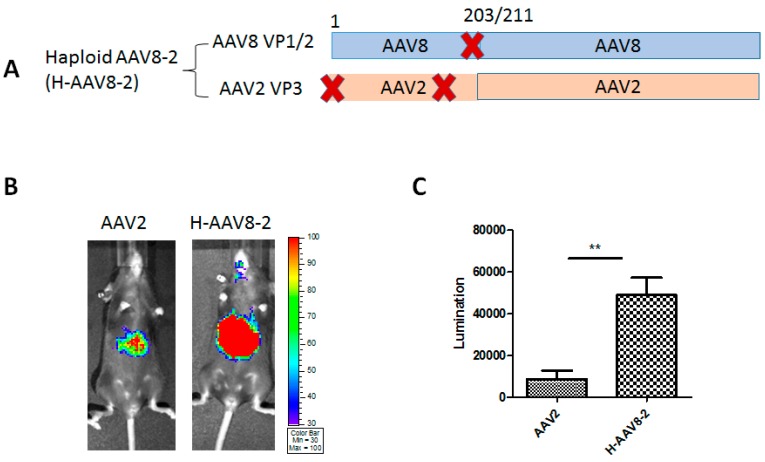
Liver transduction of haploid vector H-AAV8-2. (**A**) The composition of AAV capsid subunits. Haploid AAV viruses were produced by co-transfection of two plasmids (one encoding AAV8 VP1 and VP2, another one for AAV2 VP3). “X” represents start codon mutation. (**B**) Luciferase expression in the representative mice. 2 × 10^10^ particles of AAV vector were administered into C57BL/6. Imaging was performed at day 7. (**C**) The quantitation of liver transduction. The luciferase signal was measured by software. Each group contains five mice. Asterisks indicate a significant difference between the groups at the levels of ** *p* < 0.01.

To determine whether the components of capsid proteins could affect the transduction efficacy compared with the parental AAV2 vector, a dose of 2 × 10^10^ viral genomes (vg) of haploid AAV8-2 (H-AAV8-2)/luc (Figure 2A) or AAV2/luc virus was administered into C57BL/6 female mice via retro-orbital injection. Images were captured after 7 days using IVIS imaging system and measured by Living Imaging software. As shown in the results, the haploid vector H-AAV8-2 significantly increased the liver transduction of the mice (Figure 2B,C).

**Figure 3:**
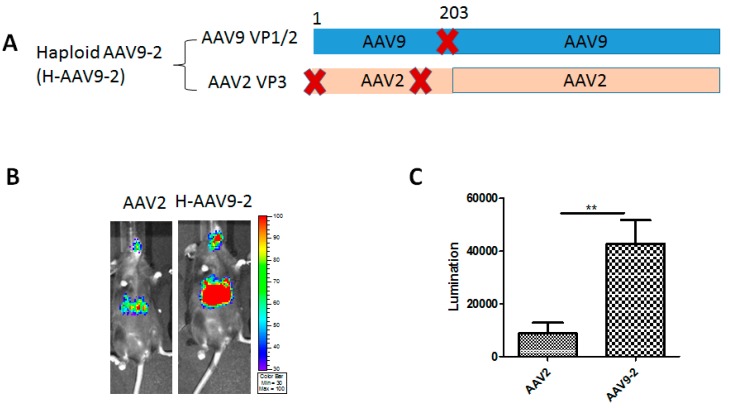
Liver transduction of haploid vector H-AAV9-2. (**A**) The composition of AAV capsid subunit. Haploid AAV viruses were produced by co-transfection of two plasmids (one encoding AAV9 VP1 and VP2, another one for AAV2 VP3). “X” represents start codon mutation. (**B**) Luciferase expression in the representative mice. 2 × 10^10^ particles of AAV vector were applied for injection. Imaging was performed at day 7. (**C**) The quantitation of liver transduction. The luciferase signal was measured by software. Each group contains five mice. Asterisks indicate a significant difference between the groups at the levels of ** *p* < 0.01.

To further test whether the VP1/VP2 derived from other AAV serotypes could enhance AAV2 transduction as well as H-AAV8-2, we produced a haploid AAV9-2 vector (H-AAV9-2) using VP1/VP2 of AAV9 and VP3 of AAV2 at the ratio of 1:1 (Figure. 3A). After 2 × 10^10^ vg of H-AAV9-2/luc vector were administered, imaging was performed at one week after vector administration. About seven-fold higher liver transduction was achieved by H-AAV9-2 compared to parental AAV2 vectors (Figure 3B,C).

**Figure 4:**
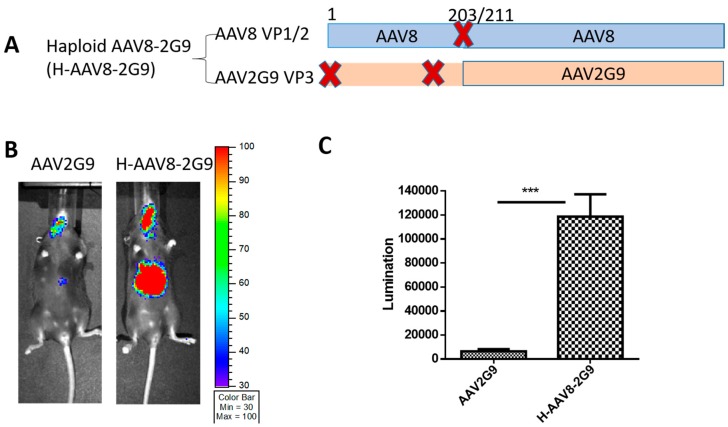
Liver transduction of haploid vector H-AAV8-2G9. (**A**) The composition of AAV capsid subunit. Haploid AAV viruses were produced by co-transfection of two plasmids (one encoding AAV8 VP1 and VP2, another one for AAV2G9 VP3). “X” represents start codon mutation. (**B**) Luciferase expression in the representative mice. 2 × 10^10^ particles of AAV vector were injected into the mice. Imaging was carried out at day 7 post vector administration, and the photon signal was measured and calculated. (**C**) The quantitation of liver transduction. The luciferase signal was measured by software. Each group contains five mice. Asterisks indicate a significant difference between the groups at the levels of *** *p* < 0.001.

It has been reported that glycan is the primary receptor of AAV9 vector [13]. The binding site of AAV9 glycan receptor has been identified. A previous study has demonstrated that when the binding residues on AAV9 virions were engrafted into the AAV2 capsid to make AAV2G9 vector, AAV2G9 was found to have higher liver tropism than parental AAV2 [14]. Herein, we produced a haploid vector (H-AAV8-2G9) with VP1/VP2 proteins of AAV8 and VP3 of AAV2G9 (Figure 4A). After H-AAV82G9 vectors were systemically injected into C57BL/6 mice, significantly higher liver transduction was observed after one week compared to that of AAV2G9 vectors (Figure 4B,C).

The above results indicate that integration of VP1/VP2 from either AAV8 or AAV9 into AAV2 VP3 virions is able to increase AAV2 transduction. Furthermore, VP1/VP2 from AAV8 was also compatible with the VP3 mutant of AAV2 to make the haploid vectors (H-AAV8-2G9) for enhanced transduction efficiency similar to other haploid vectors (H-AAV8-2 and H-AAV9-2).

### 3.2. Haploid Vector with Chimeric VP1/VP2 further Enhanced AAV2 Liver Transduction

**Figure 5:**
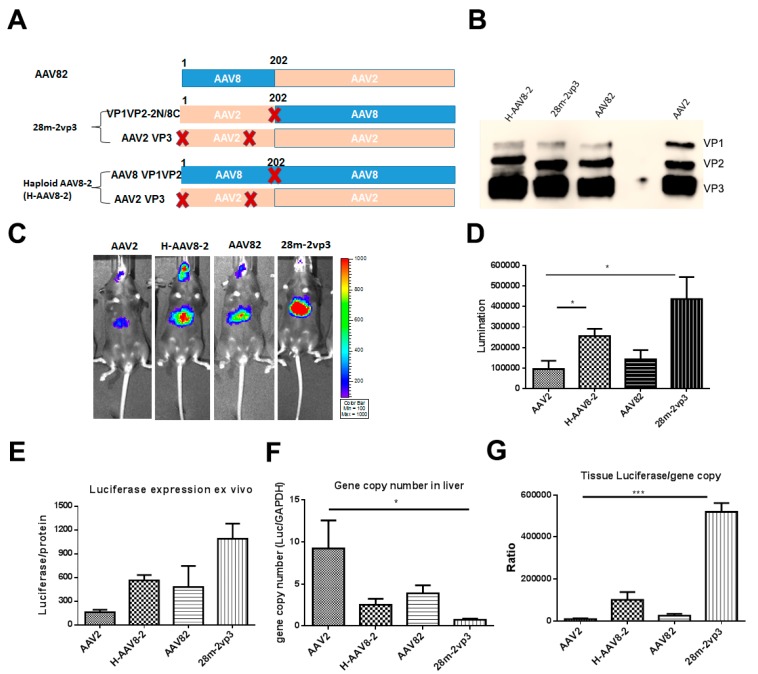
Liver transduction of haploid vectors containing the chimeric capsid components from AAV2 and 8. (**A**) The composition of AAV2 and 8 capsid subunits. Haploid AAV viruses (H-AAV8-2 and 28m-2vp3) were produced by co-transfection of two plasmids (one encoding VP1 and VP2 from AAV8, or C-terminus of VP1/2 from AAV8, VP3 from AAV2). The other haploid virus AAV82 was made by a chimeric construct with N-terminus from AAV8 and C-terminus (VP3 coding sequence) from AAV2. “X” represents start codon mutation. (**B**) Western blot of haploid AAV vectors. The same amount of haploid vector was used and B1 antibody was used as a primary antibody for detection. (**C**) Luciferase expression in the representative mice. 1 × 10^10^ particles of AAV vector were administered into C57BL/6 mice. Imaging was performed at day 7, and the luciferase signal was captured. (**D**) The quantitation of liver transduction. The luciferase signal was measured and calculated. (**E**) Ex vivo luciferase expression of the liver. (**F**) The genomic copy number of haploid virus vectors in mouse liver. (**G**) The ratio of liver luciferase expression and viral genomic copy number. Each group contains five mice. Asterisks indicate a significant difference between the groups at the levels of * *p* < 0.05 and *** *p* < 0.001.

Our previous study demonstrated that haploid vectors AAV2/8 at any ratio of AAV2 capsid to AAV8 capsid induced higher liver transduction than parental AAV2 [11]. Moreover, we also demonstrated that H-AAV8-2 (VP1/VP2 of AAV8 and VP3 of AAV2) increased liver transduction compared with that of AAV2 as described above (Figure 2). To further elucidate which AAV subunits of the individual haploid AAV2/8 vector contributes to the enhancement of transduction, we generated further two constructs. One construct was chimeric pXR82 with N-terminal of VP1/VP2 from AAV8 and the full VP3 domain from AAV2. Another one was chimeric VP1/VP2 (pXR28m) with N-terminal from AAV2 (1-202 aa) and C-terminal (VP3 domain) from AAV8 with a VP3 start codon mutation for deleting VP3 expression (Figure 5A). The chimeric pXR82 was used to make AAV82 vector, while AAV2 VP3 and pXR28m were used to generate haploid vector 28m-2vp3 (Figure 5A,B). To explore the stability of the haploid viruses, each vector was exposed to various levels of heat or low pH buffer treatment. All of the capsids of the haploid viruses were heated at 60 °C, 63 °C, and 65 °C. The results showed similar ability to react with the A20 antibody bound to intact AAV capsid, which suggests that the integration of AAV8 components into AAV2 virions did not affect the recognition of structure-dependent antibody. At 70 °C, heat treatment caused complete denaturation of the capsids among the haploid and parental vectors (Figure S1). Furthermore, the haploid viruses were also as stable as parental AAV2 vector at pH 4, 5, and 6 (Figure S2).

After the AAV vectors were injected in C57BL/6 mice at a dose of 1 × 10^10^ particles, the liver transduction efficiencies were evaluated at day 7 (Figure 5C,D). AAV82 vector (AAV82) induced slightly higher liver transduction than parental AAV2. Haploid AAV82 (H-AAV8-2) had a higher transduction than parental AAV2, which was consistent with the above results. It was noteworthy that a further increased liver transduction with haploid vector 28m-2vp3 was observed (Figure 5D). Next, we collected the livers from mice treated with AAV vectors and evaluated the luciferase expression ex vivo and the gene copy numbers in mouse liver (Figure 5E,F). The mice injected with AAV2 showed the highest viral gene copy numbers in the liver than the other groups of mice (Figure 5F), although it achieved lower transgene expression. This may have resulted from insufficient intracellular trafficking and uncoating in the nucleus. When transgene expression was normalized using genome copy number, the mice injected with 28m-2vp3 vectors had the highest luciferase expression per AAV genome (Figure 5G).

### 3.3. Haploid Vectors with the VP3 of AAV3 and VP1/VP2 of AAV8 or 9 Enhanced the Liver Transduction

**Figure 6:**
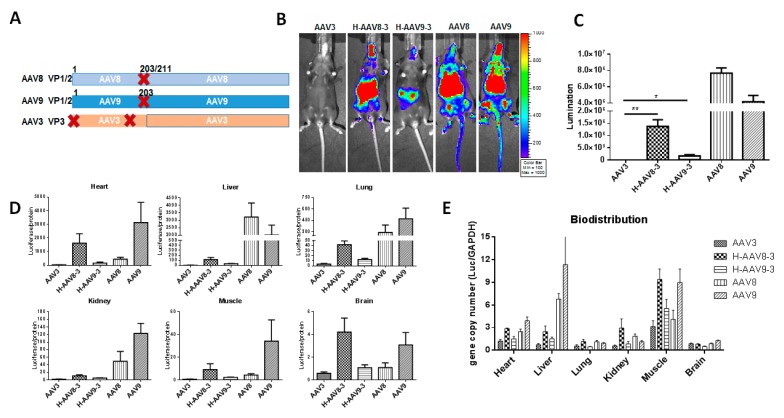
Transduction of haploid vectors with VP3 from AAV3 and VP1/VP2 from AAV8 or AAV9. (**A**) The composition of AAV capsid subunits. Haploid AAV viruses were produced by co-transfection of two plasmids (one encoding VP1 and VP2 from AAV8, or AAV9, VP3 from AAV3). “X” represents start codon mutation. (**B**) Luciferase expression in the representative mice. 1 × 10^10^ particles of AAV vector were systemically administered into the mice. Imaging was performed at day 7. (**C**) The quantitation of liver transduction. The luciferase signal was measured and calculated. (**D**) Ex vivo luciferase expression of the tissues. The harvested tissues were lysed and analyzed by luciferase assay. (**E**) Bio-distribution of haploid vectors. The AAV genomic copy number was measured by qPCR assay. Each group contains five mice. Asterisks indicate a significant difference between the groups at the levels of * *p* < 0.05 and ** *p* < 0.01.

To further investigate the compatibility and transduction efficacy of haploid vectors in which VP3 is from other serotypes and VP1/VP2 proteins are from either AAV8 or AAV9, we made an AAV3 VP3 construct which only expressed VP3 protein. Two haploid vectors H-AAV8-3 (VP1/VP2 of AAV8 and VP3 of AAV3) and H-AAV9-3 (VP1/VP2 of AAV9 and VP3 of AAV3) were produced (Figure 6A). The C57BL/6 mice received the haploid vectors H-AAV8-3 and H-AAV9-3 via systemic administration at a dose of 1 × 10^10^ vg per mouse. Imaging was carried out at day 7 after injection. Compared with AAV3, haploid vectors H-AAV8-3 or H-AAV9-3 showed higher liver transduction (Figure 6B,C), which is similar to the results obtained from the haploid vectors containing AAV2 VP3 (Figure 2 and Figure 3). However, the liver transduction of the haploid vectors was still lower than those of parental AAV8 or 9 (Figure 6B,C). Furthermore, the H-AAV8-3 vectors enhanced transgene expression in multiple other tissues, such as heart, lung, kidney, muscle, and brain when compared to transgene expression from parental AAV3 (Figure 6B,D). Notably, the tissue tropism of H-AAV8-3 was also changed in comparison to the AAV8 vector leading to higher transduction efficiencies in heart, muscle, and brain (Figure 6D). We further analyzed the viral genome copy numbers in different tissues, and found that the gene copy numbers were generally higher in heart, liver, kidney, and muscle of the mice treated with H-AAV8-3 vectors than that with parental AAV3 (Figure 6E). However, there was no significant difference in genome copy number in the heart, lung, and kidney when compared with those of parental AAV8 (Figure 6E). Interestingly, the haploid vector H-AAV8-3 induced a more peripheral tissue transduction based on the imaging profile, which is different from the results of haploid vector H-AAV8-2. The vector H-AAV8-2 only efficiently transduced into the mouse liver (Figure 2). Collectively, these results suggest that haploid vectors with VP1/VP2 from one serotype and VP3 from an alternative one were able to enhance the transduction efficiency and may change their tropism profile.

## 4. Discussion

AAV has been one of the most popular vectors for gene delivery in clinical application, and many clinical trials involving AAV vector mediated gene therapy have shown remarkable efficacy. In our previous study, we found that the haploid viruses AAV2/8 made from the co-transfection of AAV2 and AAV8 capsids enhanced liver transduction in mice when compared with the parental AAV2 or AAV8 vectors [11]. However, the mechanism of high transduction efficiency was unclear. In this study, we constructed a set of helper plasmids that expressed either VP1/VP2 or VP3 protein from different AAV serotypes. Several haploid AAV vectors were made by different combinations of these constructs and were evaluated for their transduction efficacy in mice. We found that enhancement of transduction could be achieved from haploid vectors with VP1/VP2 from one AAV vector capsid and VP3 from another one. Furthermore, our studies demonstrated that fusion of VP1/VP2 N-terminal from one serotype to VP3 virions from another serotype further increased AAV vector transduction in mice.

Previous studies have demonstrated that the capsids from two AAV serotypes were compatible enough to package haploid virions via “cross-dressing” [15]. We further expanded this concept and showed that capsid proteins from two or three different AAV serotypes (AAV2, 8, 9) were able to be assembled to generate polyploid virions [11]. Moreover, the polyploid viruses achieved higher liver transduction than parental vectors after systemic administration in mice [11]. These polyploid vectors are a mixture of AAV virions composed of different ratio of VP1, VP2, and VP3 subunits from the different serotypes in one single virion. It is impossible to elucidate which subunits play a role in the enhanced transduction of haploid AAV vectors. Earlier studies showed that VP1 played an important role for virus infectivity or stability [16,17], while both VP2 and VP3 were required for capsid formation [18]. In this study, we mutated the start codons of VP1, VP2, or VP3 to make constructs that only expressed either VP1/2 or VP3 of AAV as previously reported by other groups [18,19,20]. Consistent with the previous report [20], we found that there were weak VP3-like bands shown around the size of VP3 protein when the VP1/2 proteins of AAV8 and 9 were expressed in HEK293 cell lysis. However, these constructs did not produce virus, which was in agreement with the previous study [20]. It has been demonstrated that the virus yield of either cross-packaged AAV vectors, or haploid vectors, was similar to that of parental viruses [11,15]. In this study, no obvious difference was observed between all of the haploid viruses and parental viruses in their virus production. The AAV virion is composed of 60 subunits of viral proteins and forms a T = 1 icosahedral symmetry [21,22]. The icosahedral capsid of the virus must be stable enough for genome protection [23]. AAV capsids can be resistant to heating, and stable in a wide pH range [24]. Therefore, we explored the stability of haploid viruses through heating or low pH buffer treatment (Figures S1 and S2). According to our results, the various components of viral proteins from different serotypes did not affect the viral production or stability.

Recombinant AAV2 and 8 have been widely applied for gene delivery to the liver. AAV8 is able to deliver much more transgene than AAV2 in the livers of mice and non-human primates [25,26]. AAV9 is superior for viral genome distribution such as to the brain, heart, liver, muscle, and other tissues via systemic administration [27,28]. Our previous study indicated that either AAV8 or AAV9 with AAV2 could produce haploid vectors with a higher liver transduction than parental AAV2 [11]. Therefore, we first explored whether the haploid vectors with VP1/2 from either AAV8 or 9 and VP3 from AAV2 had high transduction efficiency in mouse liver (Figure 2 and Figure 3). The icosahedral capsid of AAV was assembled by VP1, VP2, and VP3 at an approximate ratio of 1:1:10. VP3 approximately accounts for 50 of the 60 capsid monomers, while there are only five copies each of VP1 and VP2 per capsid [29]. In this study, our results demonstrated that VP1/2 from AAV8 or 9 was embedded into AAV2 VP3 capsids, which could enhance the transduction efficacy. AAV3 vector has been studied less because of its poor transduction efficiency in mouse liver. However, recent studies have indicated that AAV3 efficiently transduced human hepatocytes [30,31]. Therefore, we attempted to make haploid viruses that were produced by VP1/2 from AAV8 or AAV9 and VP3 from AAV3 for enhancing AAV3 transduction. These two haploid vectors had higher transduction efficiency than the parental AAV3 (Figure 6). Interestingly, the tissue tropisms of the haploid AAV83 were also changed in comparison to the parental AAV8 vector, leading to higher transduction efficiencies in the heart, muscle, and brain. The transgene expression in these tissues was even higher than that of the parental AAV8. However, this was not observed in haploid AAV8-2 or AAV9-2. The mechanism for why haploid vectors with VP3 from AAV3 had differences in tropism is unknown and warrants further investigation.

It has been believed that the cell tropism and intracellular events of AAV might impact its transduction efficiency [32,33]. The AAV life cycle is complex, involving multiple steps including receptor binding, cellular entry, endosome escape, intracellular trafficking, nuclear entry, uncoating, second-strand synthesis, and so on. Intracellular trafficking and second-strand synthesis have been demonstrated as the rate-limiting steps [32,34]. A previous study reported that recombinant AAV8 appeared to traffic much more rapidly than AAV2 in hepatocytes [35]. In this study, we have attempted to explore the potential mechanisms that haploid viruses could have in significant enhancement of transduction compared with parental AAV vectors. As we know, there was a huge difference in transduction efficiency between AAV2 and AAV8 in vitro and in vivo. The recombinant 28m-2vp3 vectors displayed a lower binding ability and transduction efficiency than those of the other vectors in Huh7 cells (Figure S3). However, in mouse liver, while the gene copy number of 28m-2vp3 vectors in the liver was lower than that of the parental AAV2 vectors (Figure 5F), the highest liver transduction was achieved with the recombinant 28m-2vp3 vectors, among these four vectors. The inconsistent transduction in Huh7 cells and in mice suggests that cell lines might not be a good model to explore the mechanism of entry and intracellular trafficking steps. We speculate that the VP1/2 subunit from N-terminus of cognate VP1/2 fused with VP3 of other serotypes may better communicate with VP3 subunits for transduction enhancement.

In conclusion, the study indicated that the components of capsids from two AAV serotypes were compatible for assembly into haploid virions. VP1/2 from AAV8 or 9 combined with VP3 from AAV2 or 3 could enhance transduction efficiency of parental AAV2 or 3 in mice. Moreover, we found that the chimeric AAV2/8 VP1/2 increased transduction more than the other haploid viruses, and parental AAV2, suggesting that N-terminus of VP1/2 may need to communicate with cognate VP3 and help the intercellular life cycle of the AAV capsid. These findings not only suggest a new strategy for enhancement of AAV transduction, but also provide an insight for the basic virology of AAV.

## Supplementary Materials

The following are available online at https://www.mdpi.com/1999-4915/11/12/1138/s1, Figure S1: Binding of the monoclonal antibody to the haploid viruses after heating, Figure S2: Binding of the monoclonal antibody to the haploid viruses at the different pHs treatment, Figure S3: Transduction and binding profiles of haploid viruses in Huh7 cells, Table S1: The list of the primers.
