# Supplementary material for: Chimeric Capsid Proteins Impact Transduction Efficiency of Haploid Adeno-Associated Virus Vectors
Source: Viruses. 2019 Dec 9;11(12):1138. doi: 10.3390/v11121138 (PMC6950324; doi:10.3390/v11121138)
Supplement: Supplementary file 1 [file viruses-11-01138-s001.zip › 4-viruses-625421-suppl preint/Supplementary/Supplementary Methods Legends.pdf]

Supplementary Methods

1. AAV binding assay

Huh 7 cells were seeded at 1×10^5 cells per well in 12-well plates. Cells were infected in triplicate at a dose of 10, 000 vg per cell (vg/cell) and incubated at 4 °C for 1 hour to allow virus binding. Then, the supernatant was discarded and cells were washed 4 times using PBS. Total DNA was extracted by DNeasy Blood & Tissue Kit (Qiagen) and was detected by qPCR assay using ITR specific primer. The human GAPDH gene served as an internal control.

Supplementary Figure legends

Figure S1. Binding of the monoclonal antibody to the haploid viruses after heating. The same amount of AAV capsids were heated at 60, 63, 65, 67, or 70 °C for 10 min and then analyzed using A20 or B1 antibody by immune blot assay.

Figure S2. Binding of the monoclonal antibody to the haploid viruses at the different pHs treatment. The same amount of haploid virus particles were incubated at pH 4, 5, 6, or 7.5 for 60 min and analyzed by immune blot assay.

Figure S3. Transduction and binding profiles of haploid viruses in Huh7 cells. (A). Viral genomes after virus binding. The same amount of haploid AAV vectors were infected in Huh7 cells at 4 °C for 30 min. The media was discarded and the cells were washed. The total genomes DNA was isolated and analyzed by qPCR assay. (B). Transduction of haploid viruses. The infected cells were lysed for luciferase assay at 48 h post-transduction.
