# Supplementary material for: Chimeric Capsid Proteins Impact Transduction Efficiency of Haploid Adeno-Associated Virus Vectors
Source: Viruses. 2019 Dec 9;11(12):1138. doi: 10.3390/v11121138 (PMC6950324; doi:10.3390/v11121138)
Supplement: Supplementary file 1 [file viruses-11-01138-s001.zip › 4-viruses-625421-suppl preint/Supplementary/Supplementary Table 1.pdf]

Supplementary Table 1. The list of the primers

Production	Direction	Sequence (5'-3')
pXR2vp3	Forward	GAT TTA AAT CAG GT **CTG** GCT GCC GAT GGT TAT CTT CC
	Reverse	GG AAG ATA ACC ATC GGC AGC CAG AC CTG ATT TAA ATC
	Forward	GGA ACC TGT TAA G **GCG** GCT CCG GGA AAA AAG
	Reverse	CTT TTT TCC CGG AGC CGC C TTA ACA GGT TCC
pXR3vp3	Forward	GAT TTA AAT CAG GT **CTG** GCT GCT GAC GGT TAT CTT CC
	Reverse	GG AAG ATA ACC GTC AGC AGC CAG AC CTG ATT TAA ATC
	Forward	GAG GAA GCA GCT AAA **GCG** GCT CCT GGA AAG AAG
	Reverse	CTT CTT TCC AGG AGC CGC TTT AGC TGC TTC CTC
pXR8vp12	Forward	GTG GGA CCT AAT ACA **CTG** GCT GCA GGC GG
	Reverse	CC GCC TGC AGC CAG TGT ATT AGG TCC CAC
	Forward	CGG TGG CGC ACC A **CTG** GCA GAC AAT AAC
	Reverse	GTT ATT GTC TGC CAG T GGT GCG CCA CCG
pXR9vp12	Forward	GTG GGA TCT CTT ACA **CTG** GCT TCA GGT GGT CC
	Reverse	CC ACC ACC TGA AGC CAG TGT AAG AGA TCC CAC
VP12-2N8C	Forward	CTCTGGTCTGGGAACTAATACGATGGCTGCAGGCGGTGGCGCACC
	Reverse	GTGCGCCACCGCCTGCAGCCATCGTATTAGTTCCCAGACCAGAGG
AAVcap	Forward	CTCATTTAAATCAGGTATGGCTGCCGATGGTTATCTTCCA
	Reverse	GTTTAGCGGCCGCGACACCAAAGTTCAACTGAAACGAAT
2VP12-8VP3Mut	Forward	CTCTGGTCTGGGAACTAATACGCTGGCTGCAGGCGGTGGCGCACC
	Reverse	GTGCGCCACCGCCTGCAGCCAGCGTATTAGTTCCCAGACCAGAGG
