# Supplementary figures and images for: Chimeric Capsid Proteins Impact Transduction Efficiency of Haploid Adeno-Associated Virus Vectors
Source: Viruses. 2019 Dec 9;11(12):1138. doi: 10.3390/v11121138 (PMC6950324; doi:10.3390/v11121138)

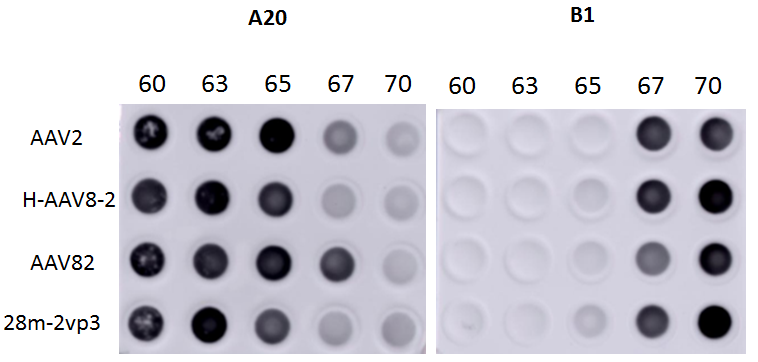

Supplement: Supplementary file 1 [file viruses-11-01138-s001.zip › 4-viruses-625421-suppl preint/Supplementary/Figure S1.tif]

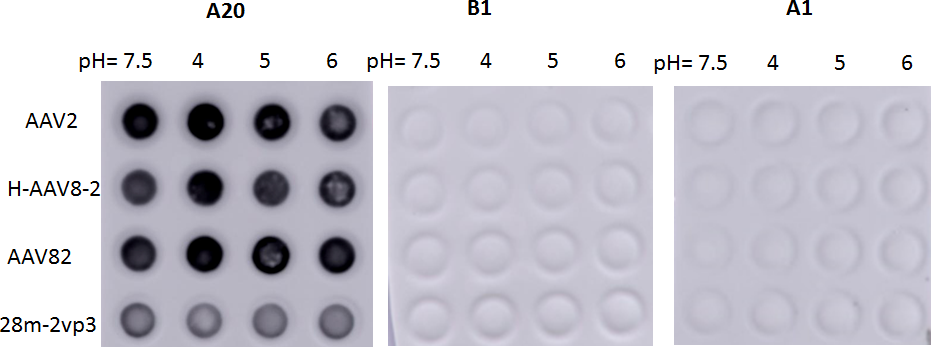

Supplement: Supplementary file 1 [file viruses-11-01138-s001.zip › 4-viruses-625421-suppl preint/Supplementary/Figure S2.tif]

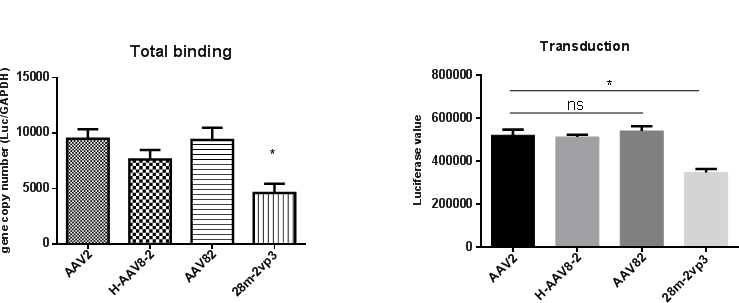

Supplement: Supplementary file 1 [file viruses-11-01138-s001.zip › 4-viruses-625421-suppl preint/Supplementary/Figure S3.tif]
